# Second External Quality Assurance Study for the Serological Diagnosis of Hantaviruses in Europe

**DOI:** 10.1371/journal.pntd.0001607

**Published:** 2012-04-03

**Authors:** Camille Escadafal, Tatjana Avšič-Županc, Olli Vapalahti, Bo Niklasson, Anette Teichmann, Matthias Niedrig, Oliver Donoso-Mantke

**Affiliations:** 1 ZBS-1, Robert Koch Institute, Berlin, Germany; 2 European Public Health Microbiology Training Programme (EUPHEM), European Centre for Disease Prevention and Control (ECDC), Solna, Sweden; 3 Institute of Microbiology & Immunology, Medical Faculty, University of Ljubljana, Ljubljana, Slovenia; 4 Department of Virology, Haartman Institute, University of Helsinki, Helsinki, Finland; 5 Apodemus AB, Stockholm, Sweden; 6 Department of Medical Cell Biology, Uppsala University, Uppsala, Sweden; Centers for Disease Control and Prevention, United States of America

## Abstract

Hantaviruses are endemic throughout the world and hosted by rodents and insectivores. Two human zoonoses, hemorrhagic fever with renal syndrome (HFRS) and hantavirus pulmonary syndrome (HPS), are caused by hantaviruses and case fatality rates have reached 12% for HFRS and 50% for HPS in some outbreaks. Symptomatic hantavirus infections in Europe are summarised as HFRS mainly due to Puumala, Dobrava-Belgrade and Saaremaa virus. While HFRS has an overall low incidence in Europe, the number of cases varies from 100 per year in all Eastern and Southern Europe up to 1,000 per year only in Finland. To assess the quality of hantavirus diagnostics, the European Network for the Diagnostics of “Imported” Viral Diseases (ENIVD) organised a first external quality assurance (EQA) in 2002. The purpose of this second EQA study is to collect updated information on the efficiency and accurateness of hantavirus serological methods applied by expert laboratories. A serum panel of 14 samples was sent to 28 participants in Europe of which 27 sent results. Performance in hantavirus diagnosis varied not only on the method used but also on the laboratories and the subclass of antibodies tested. Commercial and in-house assays performed almost equally. Enzyme immunoassays were mainly used but did not show the best performances while immunoblot assays were the less employed and showed overall better performances. IgM antibodies were not detected in 61% of the positive IgM samples and IgM detection was not performed by 7% of the laboratories indicating a risk of overlooking acute infections in patients. Uneven performances using the same method is indicating that there is still a need for improving testing conditions and standardizing protocols.

## Author Summary

Hantaviruses are endemic throughout the world and naturally hosted by rodents. The vast majority of human hantavirus infections are asymptomatic. In Europe, symptomatic hantavirus infections are summarised as hemorrhagic fever with renal syndrome (HFRS) mainly due to Puumala, Dobrava-Belgrade and Saaremaa virus. HFRS can cause fever, headache, and flank and abdominal pain. Moreover, renal dysfunction can lead to acute renal failure. Despite numerous research efforts, there is still no safe and effective vaccine or specific antiviral treatment against hantavirus infections. In this context, an accurate diagnosis as well as a reliable surveillance of hantavirus infections is essential. The diagnostics of hantavirus infections are based on serology using in-house or commercial assays. To assess the quality of hantavirus diagnostics, the European Network for the Diagnostics of “Imported” Viral Diseases organised a first external quality assurance (EQA) in 2002. In this publication we describe a second EQA study launched in 2011 with the objective to collect updated information on the efficiency and accurateness of hantavirus serological methods applied by expert laboratories. The study shows uneven performances indicating that there is still a need for improving testing conditions and standardizing protocols.

## Introduction

Hantaviruses are endemic throughout the world and naturally hosted by rodents and insectivores. Humans are mostly infected by inhalation of virus-containing aerosolized excretions (urine, saliva or feces) or bites from host rodents, and there is no transmission between humans. Two human zoonoses, hemorrhagic fever with renal syndrome (HFRS) and hantavirus pulmonary syndrome (HPS), are caused by hantavirus infections and case fatality rates can reach up to 50% for Sin Nombre and New York virus infections causing HPS and 12% for hantavirus infections causing HFRS. Nevertheless, the vast majority of human hantavirus infections are asymptomatic.

HPS are reported mainly in the Americas while symptomatic hantavirus infections in Europe are summarised as HFRS which occurs mainly due to infections by Puumala virus (PUUV) carried by *Myodes glareolus* (bank vole), Dobrava-Belgrade virus (DOBV) carried by *Apodemus flavicollis* (yellow-necked mouse) and Saaremaa virus (SAAV or DOBV-A.a) carried by *Apodemus agrarius* (striped field mouse) [1], [2], [3], [4], [5], [6]. The clinical picture is variable and depends largely on the strain of the infecting virus. HFRS is characterized by fever, acute renal failure, haemorrhage, hypotension, and vascular leakage. HFRS has a low incidence in most of Europe. Nevertheless, a survey conducted by Heyman and Vaheri in 2007, accounted for a total of 35,424 confirmed cases in all Europe. Of the total number of cases, 24,672 (70%) were reported by Finland while no hantavirus cases were reported from Spain, Italy, Cyprus or Denmark [7].

Despite numerous research efforts, there is still no safe and effective vaccine or specific antiviral treatment against hantavirus infections.

Hantavirus infections were probably highly under-diagnosed before reliable diagnostic tools became available in the 1990s. Due to the short-term and difficult detection of virus and viral nucleic acid in infected humans, the diagnostics of human hantavirus infections is mainly based on serological assays. For many years, the serological diagnosis of hantavirus infections was mainly based on immunofluorescence assays. However, in the recent years, enzyme-linked immunosorbent assays, immunoblotting, and immunochromatographic rapid tests have been developed [8]. The diagnosis is often made with in-house or commercial tests undergoing internal evaluation [9].

To assess the quality of the hantavirus diagnostics in Europe, the European Network for Diagnostics of ‘Imported’ Viral Diseases (ENIVD) (http://www.enivd.org) organised a first external quality assurance (EQA) study in 2002 with 18 laboratories participating [10]. No other EQA was performed since and little information is available concerning the overall and relative proficiency of hantavirus serology in different laboratories. For this reason, a second EQA was organised by the end of 2010 and a serum panel of 14 samples were sent to 28 participants all across Europe to be tested of the presence of antibodies.

## Materials and Methods

### Participants

A total of 28 laboratories involved in diagnostics of hantavirus infections were invited to participate in this study. Invitees are members of the European Network for the Diagnostics of ‘Imported’ Viral Diseases-Collaborative Laboratory Response Network (ENIVD-CLRN) or national/regional reference laboratories for hantaviruses or vector-borne diseases. The study was announced as an EQA study on hantavirus serological diagnostic methods proficiency, which included publishing the results in a comparative and anonymous manner.

The ENIVD-CLRN coordinated this EQA as in other previously performed EQA studies [11], [12].

### Specimen preparation

A panel of 14 samples was prepared with anti-hantavirus positive sera from seven patients infected with hantavirus diluted with fresh frozen plasma previously confirmed as negative for hantavirus. After dilution, the samples were heat inactivated (56°C, 1 h). Aliquots of 100 µl were number-coded, freeze dried for 24 h (Christ, AlphaI-5, Hanau, Germany) and stored at 4°C until dispatch.

All sera used in the panel come from an already-existing collection of patient sera from routine laboratory investigations. Sera samples were taken with the written consent from the patients and all samples were anonymized.

The proficiency panel was composed of (Table 1):

- a set of 6 positive samples consisting of serial 2-fold dilutions of a Puumala positive serum from Sweden (IgM and IgG positive)
- one serum from Slovenia positive for Puumala (IgM negative, IgG positive)
- one serum from Slovenia positive for Puumala (IgM and IgG positive)
- one serum from Slovenia positive for Dobrava-Belgrade (IgM negative, IgG positive)
- one serum from Slovenia positive for Dobrava-Belgrade (IgM and IgG positive)
- one serum from Finland positive for Puumala (IgM positive, IgG positive)
- one antiserum containing antibodies reactive for inner cell structures as specificity control
- one negative serum from Finland as negative control
- one sample with plasma used for dilution as negative control

**Table 1:** Composition of the EQA panel.

sample n°	#13	#2	#12	#6	#14	#8	#9	#11	#4	#10	#1	#3	#7	#5
**virus type**	PUU	PUU	PUU	PUU	PUU	PUU	PUU	PUU	DOB	DOB	PUU	neg	neg	neg
**dilution**	none	1∶2	1∶4	1∶8	1∶16	1∶32	1∶10	1∶10	1∶10	1∶10	none	none	1∶10	1∶10
**IgM/IgG**	+/+	+/+	+/+	+/+	+/+	+/+	+/+	−/+	+/+	−/+	+/+	−/−	−/−	−/−

PUU: Puumala; DOB: Dobrava-Belgrade; neg: negative control.

### Validation and dispatch of the panel sets

Before shipping, the serum panel was evaluated by two expert laboratories. The testing methods used included in house IFA and ELISA as well as commercial IFA.

The EQA panels were distributed to participants with full instructions. Samples were shipped by normal post at ambient temperature to the participating laboratories. We requested participant laboratories to resuspend the samples in 100 µl of water and to analyse the material as serum samples for detection of IgM and IgG antibodies against hantaviruses. They were asked to report their results and any problems encountered as well as information on the protocol details using a common formulary included in the documentation.

### Evaluation of the results

To guarantee anonymous participation, an individual numerical identification code was assigned to the results reported by each laboratory. This number was followed by a letter (A, B, C) when different data sets of results based on different methods were sent.

The results were scored in reflection of sensitivity and specificity. We assigned one point for correct virus type and one point for correct positive or negative result whereas false- negatives/positives results were not scored. Equivocal or borderline results were considered as positive. IgM and IgG results were considered separately. Data collected were entered into Microsoft Excel (Microsoft Corp., Bellingham, WA, USA).

## Results

Among the 28 invitees, the following 27 laboratories coming from 20 countries of the European region sent back their results (total of 33 data sets) and participated to the EQA:

Medical University of Vienna, Austria; Institute of Tropical Medicine, Antwerp, Belgium; Reference Laboratory for Vector-Borne Diseases, Brussels, Belgium; National Reference Vector-borne infections and leptospirosis laboratory, Sofia, Bulgaria; National Reference Laboratory for Arboviruses, Ostrava, Czech Republic; National Institute for Health Development, Virology Department, Tallinn, Estonia; Institut Pasteur, Department of Virology, Lyon, France; Institut für Medizinische Virologie, Berlin, Germany; Friedrich-Loeffler-Institut, Greifswald, Germany; EUROIMMUN AG, Lübeck, Germany; Institut für Mikrobiologie der Bundeswehr, Munich, Germany; Firm Mikrogen, Neuried, Germany; National Reference Laboratory for Viral Zoonoses, Budapest, Hungary; National Institute for Infectious Diseases, Rome, Italy; Microbiologia e Biotecnologie mediche Università di Padova, Italy; Policlinico San Matteo, Pavia, Italy; Infectiology Centre of Latvia, Riga, Latvia; Laboratoire National de Santé, Luxembourg, Luxembourg; Norwegian Institute of Public Health Department of Virology, Oslo, Norway; National Institute of Health, Águas de Moura, Portugal; Laboratory for Vector-Borne Infections and Medical Entomology, Bucharest, Romania; University of Ljubljana Medical Faculty, Ljubljana, Slovenia; Centro Nacional de Microbiología Instituto de Salud Carlos III, Madrid, Spain; Spiez Laboratory, Spiez, Switzerland; Erasmus MC Department of Virology, Rotterdam, the Netherlands; Refik Saydam National Public Health Agency, Ankara, Turkey; Reference Unit Centre for Emergency Preparedness and Response, Wiltshire, United Kingdom.

Of all data sets of results received, 46% (15/33) reported the use of enzyme-linked immunosorbent assays (EIA), 27% (9/33) immunofluorescence assays (IFA), 15% (5/33) immunoblot assays (IBA) and 12% (4/33) EIA combined EIA with IBA or IFA.

Participants used mainly commercial tests (24/33, 73%) and the remaining tests were in house methods. The performance of commercial tests was equal to that of in-house methods for both IgM and IgG detection.

Performances varied depending not only on the diagnostic method used but also on the laboratory performing the test and the subclass of antibodies detected by the test. Two out of 33 reports (6%) did not include IgM testing results and tested only for the presence of IgG antibodies by IFA (Table 2). On the other hand, all laboratories have tested for the presence of IgG antibodies (Table 3). Out of the 33 data sets obtained, about half (48%) did not report virus type specific results as they only tested for the presence or not of antibodies against hantavirus infection.

**Table 2:** EQA results with IgM detection methods.

		sample n°														
Lab n°	method	#13	#2	#12	#6	#14	#8	#9	#11	#4	#10	#1	#3	#7	#5	score
20	EIA*/IFA*	**P+**	**P+**	**P+**	**P+/−**	**P+**	**P+/−**	**P+**	**neg**	**D+**	**neg**	**P+**	**neg**	**neg**	**neg**	23
4	EIA°	**P+/D+**	neg	**P+**	**P+/−**	**P+/−**	neg	**P+/−**	**neg**	**D+**	**neg**	**P+/−**	**neg**	**neg**	**neg**	19
15 B	IFA°	**P+**	**P+**	**P+**	**P+**	neg	neg	**P+**	**neg**	**D+**	**neg**	**P+**	**neg**	**neg**	**neg**	19
1 A	EIA°	**P+**	**P+**	**P+/−**	**P+**	neg	neg	**P+/−**	**neg**	neg	**neg**	**P+/−**	**neg**	**neg**	**neg**	17
16 B	IFA°	**P+**	**P+/−**	**P+/−**	neg	neg	neg	neg	**neg**	**D+**	**neg**	**P+/−**	**neg**	**neg**	**neg**	15
24	IFA°/IBA°	**P+**	**D+**	neg	**D+**	neg	neg	**P+**	**neg**	**D+**	**neg**	**P+**	**neg**	**neg**	**neg**	15
27	IFA*	**P+**	**P+**	neg	neg	neg	neg	**P+**	**neg**	**D+**	**neg**	**P+**	**neg**	**neg**	**neg**	15
5 A	IFA°	neg	**H+**	H+	**H+**	**H+**	neg	**H+**	**neg**	**H+**	**neg**	**H+**	**neg**	**neg**	**neg**	12
12	IFA°	**H+**	**H+**	H+	H+	neg	neg	**H+**	**neg**	**H+**	**neg**	**H+**	**neg**	**neg**	**neg**	12
21	IFA°	**H+**	**H+**	H+	H+/−	**H+/−**	neg	neg	**neg**	**H+**	**neg**	**H+**	**neg**	**neg**	**neg**	12
6	EIA°/IFA°	**P+/−**	neg	neg	neg	neg	neg	**P+/−**	**neg**	**D+**	**neg**	neg	**neg**	**neg**	**neg**	11
15 A	IBA°	**P+/D+**	neg	neg	neg	neg	neg	**P+/−**	**neg**	**D+**	**neg**	neg	**neg**	**neg**	**neg**	11
16 C	IBA°	**P+**	**P+**	neg	neg	neg	neg	neg	**neg**	**D+**	**neg**	neg	**neg**	**neg**	**neg**	11
23	EIA*/IFA*	**H+**	**H+/−**	**H+/−**	neg	neg	neg	**H+/−**	**neg**	**H+**	**neg**	**H+/−**	**neg**	**neg**	**neg**	11
5 B	EIA*	**H+/−**	neg	neg	neg	neg	neg	**H+**	**neg**	**H+**	**neg**	**H+/−**	**neg**	**neg**	**neg**	9
9	IBA°	neg	neg	neg	neg	neg	neg	**P+**	**neg**	**D+**	**neg**	neg	**neg**	**neg**	**neg**	9
11	IBA°	neg	neg	neg	neg	neg	neg	**P+**	**neg**	**H+/D+**	**neg**	neg	**neg**	**neg**	**neg**	9
26	IBA°	neg	neg	neg	neg	neg	neg	**P+/−**	**neg**	**D+/−**	**neg**	neg	**neg**	**neg**	**neg**	9
28	EIA°	**H+**	**H+**	neg	neg	neg	neg	neg	**neg**	**H+**	**neg**	**H+**	**neg**	**neg**	**neg**	9
14	EIA°	**H+**	**H+**	neg	neg	neg	neg	neg	**neg**	**H+**	**neg**	neg	**neg**	**neg**	**neg**	8
7	EIA°	**H+**	neg	neg	neg	neg	neg	**H+**	**neg**	**H+**	**neg**	neg	**neg**	**neg**	**neg**	8
18	EIA°	**H+/−**	neg	neg	neg	neg	neg	**H+/−**	**neg**	**H+**	**neg**	neg	**neg**	**neg**	**neg**	8
2	EIA°	**P+**	neg	neg	neg	neg	neg	neg	**neg**	neg	**neg**	neg	**neg**	**neg**	**neg**	7
16 A	EIA°	**H+/−**	neg	neg	neg	neg	neg	neg	**neg**	**H+**	**neg**	neg	**neg**	**neg**	**neg**	7
19	EIA*	neg	neg	neg	neg	neg	neg	neg	**neg**	**D+**	**neg**	neg	**neg**	**neg**	**neg**	7
22	EIA°	**H+/−**	neg	neg	neg	neg	neg	neg	**neg**	**H+**	**neg**	neg	**neg**	**neg**	**neg**	7
1 B	EIA°	neg	neg	neg	neg	neg	neg	neg	**neg**	**H+**	**neg**	neg	**neg**	**neg**	**neg**	6
10	EIA°	neg	neg	neg	neg	neg	neg	neg	**neg**	**H+**	**neg**	neg	**neg**	**neg**	**neg**	6
13	EIA°	neg	neg	neg	neg	neg	neg	neg	**neg**	**H+**	**neg**	neg	**neg**	**neg**	**neg**	6
17	EIA°	neg	neg	neg	neg	neg	neg	neg	**neg**	**H+**	**neg**	neg	**neg**	**neg**	**neg**	6
25	IFA*	neg	neg	**H+**	neg	neg	neg	neg	**neg**	**H+**	**neg**	**H+**	**neg**	H+	**neg**	6
3	IFA*	N.A.	N.A.	N.A.	N.A.	N.A.	N.A.	N.A.	N.A.	N.A.	N.A.	N.A.	N.A.	N.A.	N.A.	0
8	IFA*	N.A.	N.A.	N.A.	N.A.	N.A.	N.A.	N.A.	N.A.	N.A.	N.A.	N.A.	N.A.	N.A.	N.A.	0

EIA: enzymatic immunofluorescence assay; IFA: immunofluorescence assay; IBA: immunoblot assay.

neg: negative result; N.A.: not available;

*: : in house assay;

**°:** : commercial assay.

P: Puumala virus; D: Dobrava-Belgrade virus; H: hantavirus.

**bold**: correct result; normal: incorrect result.

**Table 3:** EQA results with IgG detection methods.

		sample n°														
Lab n°	method	#13	#2	#12	#6	#14	#8	#9	#11	#4	#10	#1	#3	#7	#5	score
9	IBA°	**all +**	**all +**	**all +**	**all +**	**all +**	**P+**	**P+**	**P+**	**D+**	**D+**	**P+**	**neg**	**neg**	**neg**	25
11	IBA°	**P/D+**	**P/D+**	**P/D+**	**P/D+**	**P/D+**	**P/D+**	**P+**	**P+**	**H/D+**	**H/D+**	**P+**	**neg**	**neg**	**neg**	25
15 A	IBA°	**P/D+**	**P/D+**	**P/D+**	**P/D+**	**P+/−**	**P+/−**	**P+**	**P+**	**D+**	**D+**	**P+**	**neg**	**neg**	**neg**	25
15 B	IFA°	**P+**	**P+**	**P+**	**P+**	**P+**	**P+**	**P+**	**P+**	**D+**	**D+**	**P+**	**neg**	**neg**	**neg**	25
16 B	IFA°	**P+**	**P+**	**P+**	**P+**	**P+**	**P+**	**P+**	**P+**	**D+**	**D+/−**	**P+**	**neg**	**neg**	**neg**	25
16 C	IBA°	**all +**	**all +**	**all +**	**P+**	**P+**	**P+**	**P/SN+**	**P+**	**D+**	**D+**	**P+**	**neg**	**neg**	**neg**	25
20	EIA*/IFA*	**P+**	**P+**	**P+**	**P+**	**P+**	**P+**	**P+**	**P+**	**D+**	**D+**	**P+**	**neg**	**neg**	**neg**	25
6	EIA°/IFA°	**P+**	**P+**	**P+**	**P+**	**P+**	**P+/−**	**P+**	**P+**	**D+**	**H+/−**	**P+**	**neg**	**neg**	**neg**	24
26	IBA°	**P/D+**	**P/D+**	**H+**	**P+**	**P+/−**	**P+/−**	**P+**	**P+**	**D+/−**	**D+/−**	**P+**	**neg**	**neg**	**neg**	24
27	IFA*	**P+**	**P+**	**P+**	**P+**	**P+/−**	**P+**	**P+**	**P+**	**D+**	neg	**P+**	**neg**	**neg**	**neg**	23
8	IFA*	**P/D+**	**P/D+**	**P/D+**	**P+**	**P+**	**P+**	**P+**	**P+**	**D+**	neg	**P+**	**neg**	**neg**	**neg**	23
3	IFA*	**P+**	**P+**	**P+**	**P+**	**P+**	**P+**	**P+**	neg	**SE+**	neg	**P+**	**neg**	**neg**	**neg**	20
24	IFA°/IBA°	**P+**	**D+**	**P+**	**D+**	**P+**	**D+**	**P+**	**P+**	**D+**	**H+**	**P+**	**neg**	H+	**neg**	20
1 A	EIA°	**P+**	**P+**	**P+**	**P+/−**	**P+**	**P+**	**P+**	neg	**neg**	neg	**P+**	**neg**	**neg**	P+/−	18
4	EIA°	neg	**H+**	**P+**	**P+**	neg	**P+/−**	**P+**	neg	**D+**	neg	**P+**	**neg**	**neg**	**neg**	16
23	EIA*/IFA*	**H+**	**H+**	**H+**	**H+**	**H+**	neg	**P+**	**P+**	**H+**	**H+**	**P+**	**neg**	**neg**	**neg**	16
12	IFA°	**H+**	**H+**	**H+**	**H+**	**H+**	**H+**	**H+**	**H+**	**H+**	**H+**	**H+**	**neg**	**neg**	**neg**	14
14	EIA°	**H+**	**H+**	**H+**	**H+**	**H+**	**H+**	**H+**	**H+**	**H+**	**H+/−**	**H+**	**neg**	**neg**	**neg**	14
16 A	EIA°	**H+**	**H+**	**H+**	**H+**	**H+**	**H+/−**	**H+**	**H+**	**H+**	**H+**	**H+**	**neg**	**neg**	**neg**	14
19	EIA*	**H+**	**H+**	**H+**	**H+**	neg	**H+/−**	**H+**	**H+**	**D+**	**H+**	**H+**	**neg**	**neg**	**neg**	14
28	EIA°	**H+**	**H+**	**H+**	**H+**	**H+**	**H+**	**H+**	**H+**	**H+**	**H+/−**	**H+**	**neg**	**neg**	**neg**	14
5 B	EIA*	**H+**	**H+**	**H+**	**H+**	**H+**	**H+/−**	**H+**	**H+/−**	**H+**	neg	**H+**	**neg**	**neg**	**neg**	13
18	EIA°	**H+**	**P+**	**H+**	**H+**	**H+/−**	neg	**H+**	**H+/−**	**P+**	neg	**H+**	**neg**	**neg**	**neg**	13
21	IFA°	**H+**	**H+**	**H+**	**H+**	**H+**	**H+**	**H+**	**H+**	**H+**	**H+**	**H+**	**neg**	H+/−	**neg**	13
5 A	IFA°	**H+**	**H+**	**H+**	**H+**	**H+**	**H+**	**H+**	**H+**	**H+**	**H+**	**H+**	H+	H+	**neg**	12
10	EIA°	**H+**	**H+**	**H+**	**H+**	**H+/−**	neg	**H+**	**H+**	**H+**	neg	**H+**	**neg**	**neg**	**neg**	12
25	IFA*	**H+**	**H+**	**H+**	**H+**	**H+**	**H+**	**H+**	**H+**	**H+**	neg	**H+**	H+	**neg**	**neg**	12
7	EIA°	**H+**	**H+**	**H+**	**H+**	neg	neg	**H+**	**H+**	**H+**	neg	**H+**	**neg**	**neg**	**neg**	11
13	EIA°	**H+**	**H+**	**H+**	**H+/−**	**H+/−**	neg	**H+**	neg	**H+**	neg	**H+**	**neg**	**neg**	**neg**	11
22	EIA°	**H+**	**H+**	**H+**	**H+**	**H+**	neg	**H+**	**H+**	**H+**	**H+**	**H+**	H+/−	H+/−	**neg**	11
17	IFA*	**H+**	**H+**	**H+**	**H+**	neg	neg	**H+**	neg	**H+**	neg	**H+**	**neg**	**neg**	**neg**	10
2	EIA°	**P+**	neg	neg	neg	neg	neg	neg	neg	neg	neg	**P+**	**neg**	**neg**	**neg**	7
1 B	EIA°	neg	neg	neg	neg	**H+/−**	**H+/−**	neg	neg	**H+/−**	neg	neg	**neg**	**neg**	H+	5

EIA: enzymatic immunofluorescence assay; IFA: immunofluorescence assay; IBA: immunoblot assay.

neg: negative result; N.A.: not available;

*: : in house assay;

**°:** : commercial assay.

P: Puumala virus; D: Dobrava-Belgrade virus; SN: Sin Nombre; SE: Seoul virus; H: hantavirus.

**bold**: correct result; normal: incorrect result.

We can have indications on the specificity of the diagnostic methods looking at the testing results of the two negative controls and the unspecific serum. Concerning IgM antibody detection, only one false positive result was obtained with an in-house IFA in the negative control containing the plasma used for dilution (sample #7). Concerning IgG antibody detection, false positives were observed in the specificity control, sample #5 (one positive and one borderline result, 6% of all results). Surprisingly more false positives were observed among the two negative controls, samples #3 and #7 (2 positive and 2 borderline results for the dilution serum, sample #7, 12% of all results; 1 positive and 1 borderline result for the negative serum, sample #3, 6% of all results). False positives results were all obtained by commercial IFA or EIA.

To have indications on the specificity of the methods used we can also compare between the different strains of hantavirus by virus type or by place of origin. Comparing the DOBV and PUUV positive sera, we observe that the DOBV positive serum was detected more accurately by IgM detection methods than by IgG detection contrary to the PUUV sera. In fact only two of the 31 methods used for IgM detection (6,5%) have failed to detect anti-DOBV IgM while 14 of the 33 methods used for IgG detection (42%) have failed to detect anti-DOBV IgG in sample #10.

Comparing the detection of PUUV positive sera by country of origin (Sweden, Finland and Slovenian strains), no main differences in performance were observed for IgM or IgG antibody detection.

We can have indications on the sensitivity of the diagnostic methods looking at the testing results of the 6 serial dilutions of PUUV positive sera (samples #13, 2, 12, 6, 14 and 8). Regarding the testing of IgM antibodies, at least one false negative was reported by all participants except one (97%). The only method which presented no false negatives in its results was a combination of in house EIA and IFA. In contrast, IgG testing has shown to be more sensitive as half of the results for IgG detection did not report false negatives. All IBA results revealed no false negatives in IgG detection and thus showed to be very sensitive. IFA showed lower performance concerning sensitivity of IgG detection (5 tests of 9 reported false negatives, 56%) and EIA showed the lowest performance in this regard (11 tests of 15 reported false negatives, 73%). In house versus commercial assays showed similar sensitivities regarding IgG detection. Only 2 diagnostic methods (6%) failed in the detection of IgG antibodies in the highest PUUV sera dilution (sample #13) and both were commercial EIAs.

Comparing the scores obtained by the participants and the sensitivity of the tests for IgG detection, it seems that better performances were achieved by the laboratories using IBA which were all commercial assays (5 recomLine Bunyavirus IgM/IgG from Mikrogen and 1 Euroline Hantavirus profil global from Euroimmun). The first EQA study run in 2002 had shown similar good performances for commercial IBAs. No major differences were found in terms of performance concerning IgG/IgM antibody detection with IFA or EIA.

## Discussion

Although most of the participants used EIA, these tests have not shown the best performances concerning both specificity and sensitivity characteristics.

Among all participants, two have not included the detection of IgM antibodies in their routine diagnosis algorithm (6%). Furthermore the proportion of samples correctly diagnosed for IgM detection (271/434, 62%) was much lower than the proportion of samples with an accurate IgG antibody diagnosis (406/462, 88%). These elements indicate a risk of overlooking acute infections in patients with early hantavirus infections. In fact the sole presence of IgG antibodies in a serum sample could be the sign of previous contact with hantaviruses and is not enough to prove a recent infection. To confirm the diagnosis, the analysis of a second sample is required. Differences of test sensitivity depending on the antibody type detected have already been reported in the first hantavirus EQA study [10] as well as in EQA studies for the serological diagnostic of other viruses [11], [12], [13]. Nevertheless low sensitivity for IgM detection is especially observed in samples with higher dilutions of the PUUV positive serum from Sweden (samples #12, #6, #14 and #8). Therefore, the high amount of false negatives can be attributed to very low concentrations of IgM antibodies in these samples.

Regarding strain typing, it is important to point out that, because of the scoring system used in this EQA, the laboratories reporting lower scores are not necessarily the ones with lower performances. In fact, data sets reporting correct positive and negative results but not specifying the strain type obtain rather low scores although the diagnostic is entirely correct. These results are completely satisfactory in the context of clinical diagnosis as there is no specific treatment for the different hantavirus infections. The most important information is whether the patient is diagnosed positive for hantavirus or not and further analyses can always be performed. On the other hand information on the strain type is relevant for surveillance activities.

Although HFRS has a low incidence in most of Europe, the disease can be very severe. Therefore, the sensitivity of the tests used for diagnostics is more critical than its specificity. False negatives may be considered more critical than reporting a false positive as positive results can always be submitted to further testing for confirmation. In other words, in case of low disease prevalence, the predictive value of a negative test (PVN) should be higher than the predictive value of a positive test (PVP), meaning the proportion of non affected people among those tested negative should be higher than the proportion of affected people among those tested positive.

Overall, commercial and in-house assays performed almost equally. The method used (EIA, IFA or IBA) was not the main factor to have impact on the quality of the test results. From the results of this EQA, it appears clearly that the quality of the results is mostly linked to the laboratories and their use of the different protocols since their performance differ greatly even when using the same techniques. Such problems could be solved by the standardisation of the protocols and controls used and the optimisation of conditions during testing.

The previous hantavirus EQA performed in 2002 [10] also concluded that the nature of the test (in-house or commercial; IFA, EIA or IBA) used by the participants seemed to have only little influence on the performance of the diagnostic. However, IBA seemed to be slightly more sensitive than EIA and IFA. Six out of 18 laboratories participating to the 2002 EQA also took part at the second EQA (lab n°1, 10, 11, 13, 20 and 25). Two of them have improved their percentage of correct results, two of them have shown similar performance and two have decrease their performance.

Further external quality controls should be performed for hantavirus detection as EQAs are not only important for the most prevalent viral pathogens but also for rarely suspected viruses. Performing EQAs on a regular basis enables to ensure the reliability of diagnostic results, to guarantee a continuous quality of the existing diagnostic methods and further improve them.
